# Nutritional status and physical activity level as risk factor for traumatic dental injuries occurrence: a systematic review

**DOI:** 10.1111/edt.12102

**Published:** 2014-03-10

**Authors:** Marília Leão Goettems, Helena Silveira Schuch, Pedro Curi Hallal, Dione Dias Torriani, Flávio Fernando Demarco

**Affiliations:** 1 Department of Social and Preventive Dentistry, Post-Graduate Program in Dentistry, Federal University of PelotasPelotas, Brazil; 2 Department of Restorative Dentistry, Post-Graduate Program in Dentistry, Federal University of PelotasPelotas, Brazil; 3 Post-Graduate Program in Epidemiology, Post-Graduate Program in Physical Education, Federal University of PelotasPelotas, Brazil; 4 Department of Restorative Dentistry, Post-Graduate Program in Dentistry, Post-Graduate Program in Epidemiology, Federal University of PelotasPelotas, Brazil

**Keywords:** systematic review, tooth injuries, nutrition disorders, physical activity

## Abstract

**Aim:**

To systematically review epidemiological articles assessing traumatic dental injuries (TDI) rates according to the physical activity habits and nutritional status.

**Methods:**

A search was conducted using PubMed, ISI, Scopus, SciELO, LILACS, and gray literature in Brazilian Theses Databank. We searched for dental trauma, traumatic dental injuries, tooth injuries, tooth fractures, physical activity, motor activity, exercise, sedentary lifestyle, sports, obesity, body mass index (BMI), overweight, and fatness. Databases were searched in duplicate from their earliest records until 2012. Additional studies were identified by searching bibliographies of the articles. Two reviewers performed data extraction and analyzed study procedural quality using the Newcastle–Ottawa scale. PRISMA guidelines for reporting systematic reviews were followed.

**Results:**

We found 1159 articles, of whom 14 reports involving 13 studies were selected. One article was a birth cohort, one had a case–control design, and the others were cross-sectional. The quality of evidence varied across the studies and was high (9) in 3. Eleven of the studies included assessed influence of nutritional status: five show a positive association between dental trauma and overweight and six do not show any association. Regarding physical activity level, five studies assessed its effect on trauma occurrence: two detected that physical activity acts as a protective factor and two that physical active increases the risk of dental injuries, and one showed no differences in TDI occurrence. Physical activity estimated from questionnaires and BMI were the most frequently used measures, but methodological differences prevent the comparison of results.

**Conclusion:**

The results suggest that no truly causal relationship exists between dental trauma and physical activity and nutritional status. Due to the relatively low level of evidence currently present, studies with more robust design, for example, prospective cohort should address this question, especially in view of the epidemic of obesity.

Traumatic dental injuries (TDI) constitute unfortunate, painful, and distressing events with multilevel consequences, including emergency care and treatment time and costs, along with immediate and long-term emotional and social impacts 1. Although injuries have traditionally been regarded as random events, unavoidable ‘accidents, a body of knowledge now exists on the etiology of TDI 2. Some factors have been pointed out as predisposing to dental injuries, including physical characteristics, such as increased overjet and inadequate lip coverage 3,4. Demographic features, such as gender, are also associated, with boys being more affected 5,6. In addition, prevalence tends to increase with age, due to the cumulative effect of the injuries 7, leaving sequelae that cannot be fully eliminated in spite of restoration efforts 8.

One risk factor for dental injury that needs to be examined is related to childhood obesity 9. Overweight is linked to an elevated risk of non-fatal unintentional injuries 10. Available research shows contradictory results about the association between obesity/nutritional status and TDI: While obese subjects could be less exposed to traumatic injuries because they tend to be sedentary 11, other studies have suggested that the physical activity of obese children is not significantly lower than that of lean children and, for this reason, obese subjects should be more prone to injuries when falling or colliding 12–14.

The effect of physical activity habits on falls and collisions that cause traumatic dental injuries (TDI) is also controversial, similar to what occurs with nutritional status. Despite the variety of healthful benefits, vigorous physical activities also place individuals at risk for injury, including trauma to the teeth and mouth 15. Also, it has been shown that a high rate of dental injuries is caused by sports and leisure activities 16. Nevertheless, it is plausible that the practice of physical activity, being associated with improved motor skills and less probability of obesity, could be a protective factor during falls and collisions 12,17.

The knowledge of risk factors is essential for effective prevention, because it may help identify children predisposed to TDI and develop interventions to prevent dental trauma occurrence. As overweight in children is increasing and falls and collisions are the main causes of dental injuries, this association requires elucidating 18. Thus, the aim of this study was to provide an overview of the available reports on the relationship between obesity and physical activity in childhood, adolescence and/or adulthood, and dental trauma occurrence.

## Review methods

The report of this systematic review was undertaken in accordance with the Preferred Reporting Items for Systematic reviews and Meta-Analyses (PRISMA) 19 guideline.

### Inclusion criteria

The search was limited to epidemiological studies reporting etiological factors and/or the prevalence of dental trauma or risk factors for dental trauma. Studies assessing differences in dental trauma occurrence according to nutritional status and physical activity in children, adolescents, and adults were included. The study should had malnutrition/nutritional status/assessment/screening or physical activity practice/level/habits as one of the predictor variables. Studies that presented these associations were included regardless of the methodological quality. The review neither includes studies limited to groups practicing specific sports nor those assessing the frequency of sports- or leisure activities-related injuries. Review articles, case reports, and expert opinions were not included. No language or publication date restrictions were imposed.

### Information sources

Studies were identified by searching electronic databases and scanning reference lists of the selected articles. Articles published through February 2012 were considered. The databases included were the following: International Database for Medical Literature (MEDLINE)/PubMed (from 1953 to 2012), ISI Web of Knowledge (from 1900 to 2012), Scopus (from 1823 to 2012), Scientific Electronic Library Online (SciELO) (from 2000 to 2012), and Latin American and Caribbean Health Sciences (LILACS) (from 1982 to 2012). To identify relevant studies published in dissertations or theses, the database of the Brazilian Coordination of Higher Education Personnel Improvement (CAPES) was searched (from 1987 to 2012).

### Literature search

The following combination of keywords, in English and Portuguese, was used: (‘dental trauma’ OR ‘traumatic dental injuries’ OR ‘tooth injuries’ OR ‘tooth fractures’) AND (‘physical activity’ OR ‘motor activity’ OR ‘exercise’ OR ‘sedentary lifestyle’ OR sports OR obesity OR ‘body mass index’ OR overweight OR fatness).

### Study selection

Searches were carried out independently and in duplicate by two authors (M.L.G. and H.S.S.). Initially, title and keywords were read by the authors to eliminate clearly irrelevant reports. Then, the papers which full text should be obtained were selected based on the abstract reading. In case of disagreement, decisions regarding eligibility were discussed between the authors to reach consensus. If the information relevant to the inclusion criteria was not available in the abstract or if the title was relevant but the abstract was not available, the full text of the report was obtained.

### Data extraction

A data extraction sheet was developed, and two authors collected independently the information, which is shown in the Tables1 and 2. As dental trauma is a binary outcome, the summary measures collected were the risk ratio, odds ratio, and risk difference, collected in the way the authors have presented them. Also, differences in prevalence test using chi-squared and Fisher's exact tests were collected when present. The form was previously piloted. To avoid double counting, data from multiple reports of the same study were identified.

**Table 1:** Synthesis of the methods of studies that evaluated associations between dental trauma and nutritional status and/or physical activity, by study design

Author/year	Country	Sample characteristics	Instruments		
			Dental trauma	Nutritional status	Physical activity
Cross-sectional					
Petti et al./1996 29	Italy	*N *=* 824* *Age = 6–11 years* Settings: primary school	Garcia-Godoy	NA	Questionnaire concerning behavior that may predispose to injury
Petti et al./1997 12	Italy	*N *=* 938* *Age = 6–11 years* Settings: primary school	Garcia-Godoy	BMI >97th percentile of the age- and sex-specific table for the French population	Questionnaire concerning behavior that may predispose to injury
Nicolau et al./2001 18	Brazil	*N *=* 570* *Age = 13 years* Settings: private and public schools	O'Brien	BMI ≥85th percentile (>23) or BMI >2 standard deviations above the mean	NA
Nicolau et al./2003 23	Brazil	*N *=* 652* *Age = 13 years* Settings: private and public schools	O'Brien	BMI ≥85th percentile (>23), or BMI >2 standard deviations above the mean.	NA
Tapias et al./2003 30	Spain	*N *=* 470* *Age = 10 years* Settings: junior schools	WHO	BMI >85th percentile, adjusted for age 10.5 years.	NA
Malikaew/2003 26	Thailand	*N *=* 2725* *Age = 12 years* Settings: schools	Cortes	BMI in tertiles (1st tertile (<16.92); 2nd (16.92–19.62); 3rd (>16.92))	NA
Granville-Garcia et al./2006 31	Brazil	*N *=* 2651* *Age = 1–5 years* Settings: 84 state and private preschools	Hinds and Gregory	NCHS. >two Z scores for their height/weight ratio	NA
Pattussi/2006 27	Brazil	*N *=* 1302* *Age = 14–15 years* Settings: public schools	O'Brien	BMI (Cole′s criteria)	NA
Soriano et al./2007 22 and 2009 28	Brazil	*N *=* 1046* *Age = 12 years* Settings: public and private schools	Andreasen and Andreasen	BMI >97th percentile.	NA
Artun/2009 17	Kuwait	*N *=* 1583* *Age = 13–14 years* Settings: schools	NIDR index	BMI <18.50 thin and obese >30.00	Questionnaire: number of days per week with participation in physical activity
Çetinbas/2008 32	Turkey	*N *=* 2570Age = 7–9 and 11–13 years* Settings: public schools	Sweet	NA	Questionnaire: frequency of sports participation per week
Longitudinal					
Perheentupa/ 2001 24	Finland	*N *=* 5737Age=31 years* General population-based birth cohort	Questionnaire about occurrence of trauma	BMI ≥25	Questionnaire:practice of exercise that makes the person become breathless and sweat at least mildly.
Case–control					
Traebert/2002 25	Brazil	*N *=* 208 cases; 208 controls, identified in a cross-sectional study* Age = 11–13 years	O'Brien	BMI >85th percentile	NA

NA, not assessed.

**Table 2:** Synthesis of the results of studies that evaluated associations between dental trauma and nutritional status and/or physical activity, by study design

Author/year	Main findings	Quality (NOS)
Cross-sectional		
Petti et al./1996 29	(i) similar prevalence of trauma in physically and in non-physically active (OR = 0.92; 95% CI 0.59–1.42)	4
Petti et al./1997 12	(i) higher prevalence of trauma in obese children (OR = 1.45; 95% CI 1.08–1.94) (ii) physical activity was a protective factor (OR: 0.50;95% CI 0.38–0.67)	7
Nicolau et al./2001 18	(i) higher prevalence of trauma in overweight children (OR = 1.93; 95% CI 1.10–3.38)	8
Nicolau et al./2003 23	(i) similar prevalence of TDI among overweight adolescents and non-overweight (OR 1.43; 95% CI 0.85–2.41)	9
Tapias et al./2003 30	(i) similar prevalence of TDI among overweight and non-overweight(OR 0.66; 95% CI 0.39–1.13)	8
Malikaew/2003 26	(i) similar prevalence of trauma in the 3 BMI categories: 1st 32.30%; 2nd 37.30%; 3rd 35.40% (*P *=* *0.09)	8
Granville-Garcia et al./2006 31	(i) overweight/obese children exhibited greater chance of suffering trauma than those without overweight (OR = 2.50; 1.89–3.30)	7
Pattussi/2006 27	(i) obesity was not associated with dental injury neither in boys nor in girls.	9
Soriano et al./2007^22^ and 2009 28	(i) obese subjects sustained more traumatic dental injuries than non-obese subjects (OR = 1.84; 95% CI = 1.02–3.33)	8
Artun/2009 17	(i) no difference was detected in injury rate among the subjects in the three BMI categories. (ii) trauma was more prevalent among those participating in sports (OR = 1.64; 95% CI 1.23–2.17)	5
Çetinbas/2008 32	(i) children who practiced sports once a week had lower sports-related fractures (7/1693) than those associated with sports activities 1–3 (14/733) and 4–6 (6/132) days a week (*P *<* *0.05).	6
Longitudinal		
Perheentupa/2001 24	(i) overweight was a risk factor for dental fractures (RR = 1.10; 95% CI 1.04–1.18) and for displacements/avulsions (RR 1.25; 95% CI 1.10–1.43) (ii) physical activity was not associated with displacements or avulsions (RR 0.95; 95% CI 0.77–1.17); those who had physical activity 1–3 times week^−1^ had lower risk of dental fractures (RR 0.93; 0.87–0.99) than those who practiced 0–3 times month^−1^	6
Case–control		
Traebert/2002 25	(i) TDI was not associated with BMI (OR = 1.03; 95% CI 0.58–1.82)	9

NOS, Newcastle–Ottawa scale; ranges from 0 to 9; TDI, traumatic dental injury.

### Assessment of study quality

Two independent reviewers evaluated the quality of the studies that met the eligibility criteria using the Newcastle–Ottawa Quality Assessment Scale (NOS) for cohort/case–control studies. Cross-sectional studies were evaluated using the modified Newcastle–Ottawa scale 20. The assessment of bias included the following items: (i) selection of the study population, (ii) comparability of subjects, and (iii) assessment of exposure or outcome, according to the study design. According to these criteria, study quality was rated on a scale from 1 (very poor) to 9 (high). Disagreements were resolved by consensus. A study could receive 1 point per item for (i) selection of the study population (0–4 items), (ii) comparability of subjects (0–2 items), and (iii) outcome for cohort and cross-sectional studies (0–3 items).

## Results

### Study selection

**Fig 1:**
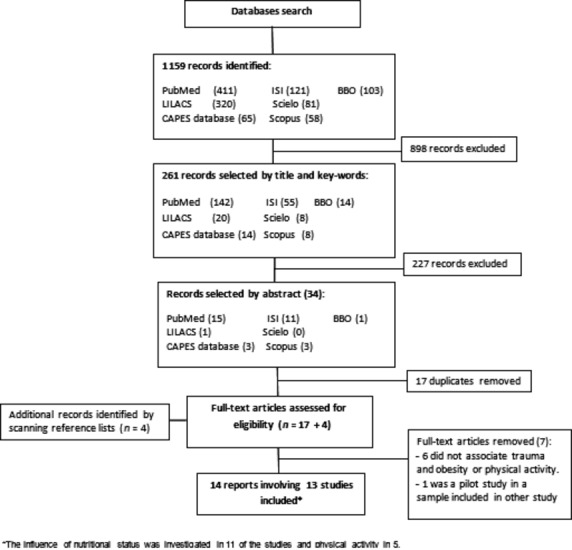
Selection process for studies evaluating the association between dental trauma and nutritional status and/or physical activity.

Figure1 shows a flowchart outlining the number of articles identified at each step of the literature search. The search at databases provided a total of 1159 citations. The screening process of the title identified 261 articles, which met the inclusion criteria. Of these, 227 studies were discarded after reviewing the abstract. After adjusting for duplicates, 17 remained and the full text versions of these publications were then retrieved and their reference lists screened for further relevant publications. Four relevant articles were then identified. The full text of the articles was examined, and a total of 14 publications involving 13 studies met the inclusion criteria and represent the total data set included in this structured review. Two of them 21,22 performed different analyses in the same sample and were accounted for one, whereas two other studies 18,23 had used the same population, but included different sample sizes and had controversial results and thus were included as two different studies.

### Study characteristics

We have decided not to perform a meta-analysis because the designs of the studies were too different, the outcomes measured were not sufficiently similar, due to concerns about the quality of the studies. Thus, we report a qualitative synthesis of the results: Table1 shows study characteristics, and Table2 presents a synthesis of the results of the selected studies correlating TDI and nutritional status and/or physical activity level.

Of the 13 studies, one was conducted in a birth cohort 24, one had a case–control 25 design, and the others were cross-sectional. The sample sizes have varied considerably, from 416 to 5737. The cohort 24 and the case–control 25 study presented sample size calculations, as well as 4 of the 11 cross-sectional studies 22,23,26–28. Eleven of the studies included assessed influence of nutritional status and five assessed physical activity level. These studies were published between 1996 and 2009. Of the studies evaluated, only two were published in the 1990s, and nine papers were published in the last 10 years. Of the 13 studies, six were performed in Brazil, two in Italy, and the others in Spain, Finland, Thailand, Kuwait, and Turkey.

In the case–control study included, Traebert et al. 25 considered the cutoff point for obesity BMI scores equal or above the 85th percentile. The study found no association between BMI groups and dental trauma between 11- to 13-year-old adolescents, using univariate regression analysis (*P* = 0.09) and analysis of conditional multiple regression (*P* = 0.92).

A large follow-up evaluation of a population-based birth cohort in Finland 24 concluded that an increased lifetime prevalence of dental trauma at 31 years of age was associated with overweight (OR = 1.1; 95% CI = 1.04–1.18) and that regular physical activity decreased tooth fracture occurrence (OR = 0.93; 95% CI = 0.87–0.99). When the genders were compared, a high BMI increased the risk among females only. In this study, BMI of 25 and over was considered overweight and physical activity consisted of a questionnaire to assess frequency of regular exercise that makes the person become breathless and sweat at least mildly.

The earliest published study with a cross-sectional design included in this review was carried out in 1996. Petti and Tarsitani 29, evaluated the difference in the prevalence of TDI between physically active schoolchildren aged 6 to 11 years and non-physically active, classified by means of a questionnaire administered to subjects and parents concerning children's behavior which may predispose to injuries such as bicycle riding, skating, skateboarding, and contact sport. The authors found that the prevalence of trauma was 19.34% (*n *=* *61) in physically active children and 20.78% (*n *=* *106) in non-physically active (*P *=* *0.35).

One year later, the same authors 12 found that children above or equal to the BMI value of the 97th percentile of the age- and sex-specific reference table for the French population had a 31.87% prevalence of dental trauma while non-obese children had a 20.07% prevalence (*P* < 0.01; OR = 1.45; 95% CI = 1.21–2.33). Besides this, the authors detected a difference in severity of the injuries: While obese children presented mostly mild trauma, occurred indoor (38.27%), non-obese children, despite the lower prevalence, had more severe traumas that occurred outdoor (33.77%). Physical activity level was also assessed by means of a questionnaire including behavior that may predispose to injury. As six of eight questions concerned the children's lifestyle, the trauma predisposition score was also considered an estimate of physical activity. In the multiple logistic regression, they observed that the more active children presented lower dental trauma occurrence (*P *<* *0.01; OR = 0.50; 95% CI = 0.38–0.67).

Using a different cutoff point for obesity, Nicolau et al. 18 conducted a study with 13-year-old adolescents in public and private schools in Brazil and found that those who had BMI scores equal or above the 85th percentile had a 28.8% 23 prevalence of dental trauma, compared with 18.4% (90) in non-overweight. In the adjusted analysis, overweight children were more inclined to injuries (OR = 1.93; IC 95% = 1.10–3.38).

In 2003, Nicolau et al. 23, analyzing 652 Brazilian adolescents (13 years old), showed that boys (27.5%) and overweight adolescents (25.8%) had more dental injuries than girls (13.4%) and non-overweight adolescents (19.3%). However, the relationship between overweight and dental trauma was not statistical significant (*P *=* *0.098).

Another cross-sectional study carried out in 2003 with 2725 schoolchildren aged 12 years was the only one of the reports assessed in this review that used the Cortes’ criteria (2001) to evaluate of dental trauma. To measure the nutritional status of the sample, the authors used the BMI in tertiles. Despite the extensive evaluation, the study was not able to detect association between nutritional status and dental trauma (*P *=* *0.09) 26.

In the same year, Tapias et al. 30, studying schoolchildren aged 10 years in Spain, observed that, among the children who had dental trauma, a total of 29.3% were overweight. In the children without dental trauma, 36.9% were overweight. In the logistic regression, the relationship between these characteristics was not confirmed.

Another cross-sectional study in Brazil conducted by Granville-Garcia et al. 31 with 2651 preschool children evaluated the relationship between obesity and the occurrence of dental trauma. In this study, the authors used the National Center for Health Statistics (NCHS) standard, which is preconized by the World Health Organization (WHO), adopting the *Z* score as baseline. In the multiple logistic regression analysis, they observed that overweight/obese children, considered those above 2 *Z* scores for their height/weight ratio, exhibited 2.5 times (*P *<* *0.001; OR 2.5; 95% CI = 1.89–3.30) greater chance of suffering trauma when compared to those without overweight/obesity.

The study conducted by Patussi et al. 27 in 2006, also in Brazil, with a randomly selected sample of adolescents found that obesity, assessed by Cole's criteria, was not associated with dental injury either in boys (*P *=* *0.388; OR = 1.26; 95% CI = 0.74–2.16) or in girls (*P *=* *0.944; OR = 1.02; 95% CI = 0.52–1.87).

In the studies of Soriano et al. 22,28, the association between obesity and higher frequency of dental trauma among 12-year-old adolescents was statistically significant (*P *<* *0.05; OR = 1.84; CI = 1.02–3.33). When differences were compared by type of school 22 and by sex 28, the influence detected for the whole sample was not present.

Çetinbas et al. 32 evaluated the physical activity among 2570 children in public schools in Brazil according to the practice of sports per week and found that children who practiced sports once a week had significantly lower fracture rates than those associated with sports activities 1–3 and 4–6 days a week (*P *<* *0.05); however, there was no statistical difference between 1–3 and 4–6 days of sports participation (*P *>* *0.05).

Artun et al. 17 evaluated a sample of 1583 adolescents in Kuwait and detected an effect of participation in sports and other physical activities in the whole sample in the regression analysis, but the number of days per week with activity had no effect, and increased physical activity was not included in the final prediction model. Moreover, the difference in dental trauma among those who were and were not physically active (17.6% vs 11.5%) could not explain the gender difference in injury rate of 19.3% vs 9.7% 4 of the subjects, because no effect was detected of increased physical activities on dental trauma in each subsample of boys and girls, despite the large gender difference in physical activities.

### Quality assessment

Regarding quality, in a scale that ranges from 1 (very poor) to 9 (high quality), cross-sectional studies received between four to nine points (Table2), with an average of 7.2. One retrospective cohort study received the score of six points and a case–control study received nine points. Lower scores were mainly related to the lack of representativeness of the sample, lack of a multivariate analysis, and non-response rate. The studies that found association of TDI with nutritional status had scores ranging from 6 to 8; studies that detected association with physical activity had scores ranging from 5 to 7. Among the studies that achieved 9, the maximum score, none have detected association 23,25,27.

## Discussion

Systematic reviews may make clear the strength of evidence on factors associated with dental trauma 33. This information is essential for planning dental health education programs aimed to reduce the incidence of TDI. This systematic review involved the search of multiple electronic databases, and the reference lists of literature reviews were searched for other studies that could also be included.

The review detected that, regarding the effect of nutritional status, five of 11 studies showed the influence of overweight/obesity on dental trauma occurrence 12,18,22,24,28,31, while the others did not detect any statistically significant relationship between obesity and TDI 17,23,25–27,30. One of the studies was conducted with preschool children 31 and one assessed the influence of weight on dental trauma in adults 24. In both studies, obese individuals presented increased risk of dental injuries. Among the nine studies assessing schoolchildren and adolescents, three of them detected the influence of nutritional status on dental trauma occurrence. No study found that obese individual, being sedentary, would present lower prevalence of dental trauma. A possible explanation to the detected association is that overweight children tend to present less agility, skillful, and dexterous, which could make them more prone to accidents and, consequently, to dental trauma. Differences in injury severity were investigated in one study 12, and the authors concluded that obese children are exposed to dental trauma at higher frequency but mostly suffering slight injuries, because they tend to stay on their own and play mostly at home. Few studies had performed sex-stratified analyses 24,27,28, one of which detected that a high BMI increased the risk among females only 24.

The influence of physical activity level was included in only five studies, and the effect of increased physical activity and participation in sports as a risk factor for dental trauma is controversial: a similar number of studies were found for and against the association. One of them showed no differences among individuals considered actives and individuals inactive 29. Two of the studies 12,24 found an inverse relationship between dental injuries and an active lifestyle, demonstrating that regular physical activity seemed to decrease the risk for dental fractures. This is possibly connected with the fact that physical activity is conductive with improved motor skills and increased self-confidence 34–36. Subjects frequently playing sports and lively games would be not only less obese but also more skillful and, for this reason, less prone to trauma when they fall or sustain impacts during interaction and play with family and friends 12. Also, another study concluded that athletic activities are not dangerous from the point of view of dental trauma, because few episodes of dental trauma occurring were found during these activities 37. In opposite, Artun et al. 17 and Çetinbas 32 observed higher occurrence of dental trauma in those individuals participating in sports.

Some reasons for the detected inconsistencies may be proposed, ranging from methodological aspects to quality of the studies. The Newcastle–Ottawa scale has been used to assess quality and it is able to standardize the scores among all studies. The quality of the studies ranged between four and nine points, which demonstrate methodological variability. This scale was developed to assess the quality of non-randomized studies with its design, content, and ease of use directed to the task of incorporating the quality assessments in the interpretation of meta-analytic results. Thus, some studies have adopted cutoff point to classify studies as high or low quality, as compared to the other studies included in the review. In the present study, six of the studies had a score of 7 or less and seven studies had score of 8–9. Considering only the high quality studies, among those with eight points, two have detected association between dental trauma and obesity whereas two did not. Among those obtaining score 9, no association was detected. Lack of adjustment for possible confounders and lack of representativeness of the sample were observed in most of the studies. It should be emphasized that future studies should adopt multivariate analysis and investigators need to provide suitable calculations to ensure that a study is capable of detecting real associations between study factors 38. The sample size calculation involves the application of a series of mathematical formulae that have been designed to ensure precision in estimating the population parameters or to obtain significant results in those studies comparing groups 39.

To help establish causality, the findings were discussed according to Hill's criteria of causation 40. Regarding temporality, children must first be obese and then increase their risk of dental trauma, for example. This is probably the case. Evidence for plausibility is the fact that overweight is linked to an elevated risk of non-fatal unintentional injuries 10. Experimental evidence for the associations exists, as previous study, conducted in a Brazilian city, has diagnosed fewer children with dental trauma in health promoting schools, demonstrating that the commitment toward health and safety has improved oral health outcomes 41.

To adequately assess the mentioned association, studies should use an appropriate design. The findings demonstrate a considerable tendency toward conducting and publishing cross-sectional studies. The only longitudinal study included has enquired about the lifetime occurrence of injuries and compared with the habits at the time of data collection. This may represent a limitation, as injuries have a cumulative effect and might have occurred anytime after tooth eruption and physical activity and nutritional status may not correspond to that moment. Thus, future research should also correlate determinants prospectively.

Regarding strength of the association, the hypothesis stating that a causal association exists between nutritional status, physical activity, and dental trauma is not strongly reinforced by the results: Significant outcome measures have varied from 1.10 to 2.5 for the association with overweight and from 0.50 to 1.64 for physical activity.

Regarding to the dose–response relationship criterion, it can be noted that the cutoff point adopted for obesity can influence the detected association. Although most studies used BMI, two studies used the National Center of Health Statistics (NCHS) tables 22,28,31. Among studies that used BMI, one possible reason that explains differences in results is the cutoff point adopted. In the study of Petti et al. 12, children whose BMI was above or equal to the 97th percentile had a 1.45 higher chance of suffering trauma. Noteworthy, the high cutoff point may have influenced results. One study used tertiles to consider BMI categories and failed to detected association. Among studies using the 85th percentile to consider obesity, just 1 detected association 18 and 3 did not 23,25,30. Patussi 27 adopted Cole′s criteria and was not able to detect association. Results of the studies assessing physical activity cannot be directly comparable, because of the difference in instruments for measurement used. There are a wide number of measures to assess physical activity level 42, but none of the studies have used a validated tool. Whereas some studies considered only frequency of sports participation per week 17,32, another considered the frequency of exercise that makes the person become breathless and sweat 24 and one assessed trauma predisposition by means of a questionnaire with eight questions, six of which concerning the children's lifestyle 12. Considering that measurement instrument is an important methodological aspect, it is important that further studies should use a validated tool to assess differences in trauma occurrence between sedentary and active individuals.

The consistency of the findings of available studies is not absolute. Similar findings have been observed between different populations and in different times 17,26, but a trend cannot be observed 12,29. The high prevalence of traumatic dental injuries implies that adequate preventive programs at a population level should be carried out. Also, the prevalence of overweight and obesity has reached epidemic levels 43, being considered an important public health problem, with several consequences and significant costs. In addition, high rates of sedentary lifestyle are still observed worldwide, in spite of all accumulated scientific knowledge on the benefits of physical activity for health 42. As a number of diseases share these risk factors, a common risk factor approach is suggested to improve child's health.

## Conclusions

The differences in relation to the methodologies used and the small number of published articles, especially for adults and preschool children, hinder the comparison of the studies. Results show the need of studies adopting appropriate methods in this area, with more robust design, to allow the truly determination of the potential causal relationship between nutritional status and physical activity with the outcome (dental trauma). Despite the fact that a positive association of obesity and dental trauma was detected, the available literature data could not allow to conclude about this causal relationship. Future research should include representative samples, to use validated measurement tools and adjust for possible confounders. Studies should also use appropriate design, for example, prospective cohort, that allows correlating determinants prospectively. Longitudinal studies of incidence cases are important to assess the effect of nutritional status and physical activity level on dental trauma occurrence.
